# Special Issue “Metal and Metal Oxide Nanoparticles: Design, Characterization, and Biomedical Applications”

**DOI:** 10.3390/ma14237287

**Published:** 2021-11-28

**Authors:** Tania Limongi

**Affiliations:** Department of Applied Science and Technology, Politecnico di Torino, Corso Duca degli Abruzzi 24, 10129 Turin, Italy; tania.limongi@polito.it

The current Special Issue entitled “Metal and Metal Oxide Nanoparticles: Design, Characterization, and Biomedical Applications” aims to present contributions from all scientists producing and/or applying metal and metal oxide nanoparticles in a diagnostic, therapeutic or theranostics context.

Developing new materials is usually a time-demanding and meticulous process, but at the same time, it is one of the more promising solutions to obtain a cleaner, safer, and smart future. In more detail, when referring to nanomaterials, an increasingly successfully tool of nanotechnologies, nanoparticles are categorized as materials in which at least one dimension is less than 100 nm in diameter. Among the various nanoparticle categories, metal and metal oxide nanoparticles stand out as emerging nanotechnological solutions for a wide range of biological and medical physio/pathological open questions [1,2,3]. This Special Issue covers the fundamental science, design, characterization, and biomedical applications of metal and metal oxide nanomaterials and the five papers here presented embrace all the aspects determining the performance of these systems, ranging from their synthesis, design, chemical, physical, and biological functionalization, to their characterization and successful applications.

Metal-based nanoparticles (MNPs) include metal NPs, metal oxide NPs, quantum dots (QDs) and magnetic NPs and, thanks to their chemical physical properties, have gained much traction for their functional use in biomedicine.

In the last decades, many authors have described how the use of MNPs in stimuli-responsive systems design encourages their clinical translatability, improving also their preclinical investigation [4,5,6,7,8]. In their review, Caneparo et al. highlighted how reactive oxygen species (ROS) generation could be considered an effective nanotechnology tool for alternative therapies, such as photodynamic therapy (PDT), high-intensity focused ultrasound therapy (HIFU), photothermal therapy (PPT) and sonodynamic therapy (SDT) [9].

Laurenti et al. explored the use of a new composite material based on the incorporation of mesoporous flower-like ZnO micropowders into polyHEMA and poly(HEMA-co-AA) hydrogels for drug eluting stent applications. Their release study pointed out that the poly(HEMA-co-AA)@ZnO_0.1% formulation satisfied the multifunctional requirements needed in the field of ureteral stent applications such as antibacterial effects, drug elution and biodegradability [10].

Bartoli et al., summarizing in their review the complex chemical behavior of bismuth during the transformation of its compounds to oxide and bismuth oxide-phase transitions, reported the main achievements reached by using nanostructured bismuth oxide and related nanomaterials for the design and the application of new diagnostic and theranostic tools [11].

Svensson et al. in their communication, reported a proof of principle for the self-assembly of titania nanoshells from anisotropically functionalized titania NPs successfully utilized for fast-release drug delivery application [12].

To conclude, Mylkie et al. described the efficacy of biocompatible natural polymer-coated magnetite NPs for protein immobilization, highlighting their application in biomedicine [13].
